# Large-scale forecasting of *Heracleum sosnowskyi* habitat suitability under the climate change on publicly available data

**DOI:** 10.1038/s41598-022-09953-9

**Published:** 2022-04-12

**Authors:** Diana Koldasbayeva, Polina Tregubova, Dmitrii Shadrin, Mikhail Gasanov, Maria Pukalchik

**Affiliations:** 1 Center of Life Sciences, Skolkovo Institute of Science and Technology, Moscow, Russian Federation 121205; 2 RAIC, Skolkovo Institute of Science and Technology, Moscow, Russian Federation 121205; 3 Irkutsk National Research Technical University, Irkutsk, Russian Federation 664074; 4 Digital Agriculture Laboratory, Skolkovo Institute of Science and Technology, Moscow, Russian Federation 121205

**Keywords:** Environmental impact, Ecological modelling, Invasive species

## Abstract

This research aims to establish the possible habitat suitability of *Heracleum sosnowskyi* (*HS*), one of the most aggressive invasive plants, in current and future climate conditions across the territory of the European part of Russia. We utilised a species distribution modelling framework using publicly available data of plant occurrence collected in citizen science projects (*CSP*). Climatic variables and soil characteristics were considered to follow possible dependencies with environmental factors. We applied Random Forest to classify the study area. We addressed the problem of sampling bias in CSP data by optimising the sampling size and implementing a spatial cross-validation scheme. According to the Random Forest model built on the finally selected data shape, more than half of the studied territory in the current climate corresponds to a suitability prediction score higher than 0.25. The forecast of habitat suitability in future climate was highly similar for all climate models. Almost the whole studied territory showed the possibility for spread with an average suitability score of 0.4. The mean temperature of the wettest quarter and precipitation of wettest month demonstrated the highest influence on the HS distribution. Thus, currently, the whole study area, excluding the north, may be considered as s territory with a high risk of HS spreading, while in the future suitable locations for the HS habitat will include high latitudes. We showed that chosen geodata pre-processing, and cross-validation based on geospatial blocks reduced significantly the sampling bias. Obtained predictions could help to assess the risks accompanying the studied plant invasion capturing the patterns of the spread, and can be used for the conservation actions planning.

## Introduction

The relocation and introducing of alien species into new habitats are recognised as one of the major drivers of global biodiversity loss^1–3^. Invasive alien (non-indigenous) species *IAS* tend to spread rapidly and pose a serious threat to endemic species due to e.g. the competition in the resource use, allelopathy occurrence, toxicity of IAS^4,5^. Thus, the emergence of IAS can dramatically change the functioning of the natural communities and overall ecosystem structure^6–8^.

Such common occurrences as human living territory expansion, globalization of transport, and changing of the land-use types favor species invasion. With that, the estimated costs of the elimination of IAS are usually quite high. The specific of individual IAS limits the implementation of such practices. The other constraints are the territory’s size that needs to be treated, the possibility of negative side outcomes because of the use of chemical and biological control agents, and the development of the invasion process^9–11^. IAS disproportionally affect the most vulnerable communities in poor areas, at the locations of abandoned and disturbed lands. Thus, their spread is clearly pulling up the achievement of the Sustainable Development Goals^12^.

*Heracleum sosnowskyi* Manden (*Hogweed, HS*) is one of the examples of extremely dangerous invasive species. The natural habitat of HS is the central and eastern Caucasus area and adjacent regions, Transcaucasia region and Turkey^13^. Large biomass and the ability to live and develop in cold climates became HS a popular crop in agriculture in the middle of the 20th century^14^. However, soon the unpleasant odor of milk and meat of animals that were fed with HS fodder and the phototoxic effect of above-ground parts of HS were revealed, and as a result, the cultivation was abandoned.

The need to forecast the potential extinction of different species in different spatial and temporal contexts, has led to the Species Distribution Modelling (*SDM*) development. SDM framework is based on the ecological concept assuming that the distribution of species is explained by the set of factors, such as environmental requirements and interactions with other living organisms, physiology characteristics, evolution history^15,16^. General workflow of correlative SDM consists of (1) obtaining the data about the species of study occurrences: presence-only data, presence/absence data, abundance data; environmental characteristics data, sometimes considering biotic interactions as well, (2) search of the interconnections between these data, and (3) building the map of predicted distributions across the region of interest. SDM framework is implemented in a variety of packages and libraries in most common programming languages, such R or Python, and allows to use several different statistical or machine learning (ML) models, e.g., generalized linear models, classification and regression trees, random forest (RF), support vector machine, artificial neural networks, and others, and ensemble of them^17–21^. In terms of data availability these models mostly differ from each other by the requirements to occurrence records, i.e., should the occurrence data be represented by two classes—presence and absence, or it can be only presence data^22^. The choice of the appropriate modelling method significantly affects outcomes and depends on multiple factors: size of the territory of study, type of the environment considering its changing dynamics, characteristics of modelling species, data availability, while it has become more popular to use ensembles-across-methods forecasting^23^. However, there are no strict directives on how to implement the ensemble, e.g. should one estimate an average prediction or weighted average prediction—thus, this solution is not so straightforward in comparison with basic modelling methods^24^. Some studies demonstrate higher performance of a particular model above others for specific cases. For example, it has been shown that RF approach is highly suitable for forecasting on large territories with a limited amount of data^25^, while for marine environments, ensemble models are recommended to use^26^.

Correlative SDM has a conceptual limitation—it is assumed to capture realized ecological niche, which is confusing when IAS is the object of the study^27^. Another struggle is the quality of using data, precisely, the occurrence and absence of the species. It is stated that pseudoabsence data should be field corrected, otherwise it shows strong bias, decreasing the species prediction perfomance^28^. In reality, such correction is almost impossible for large territories and requires significant collections of remote sensing data with appropriate resolution. It is much more controversial issue when the spread of IAS is the case. In case of a sufficient number of verified absence points of the studied IAS, a question that remains: is this location unsuitable indeed for the selected IAS, or the IAS has not reached it yet^29^. However, despite all the mentioned limitations, correlative SDM still is the primary tool for the IAS distribution modelling^30^. Another possibility is to use mechanistic SDM, which is developed on the process-based approach, e.g. phenology model^31^, but such models require calibration of many internal parameters.

**Figure 1:**
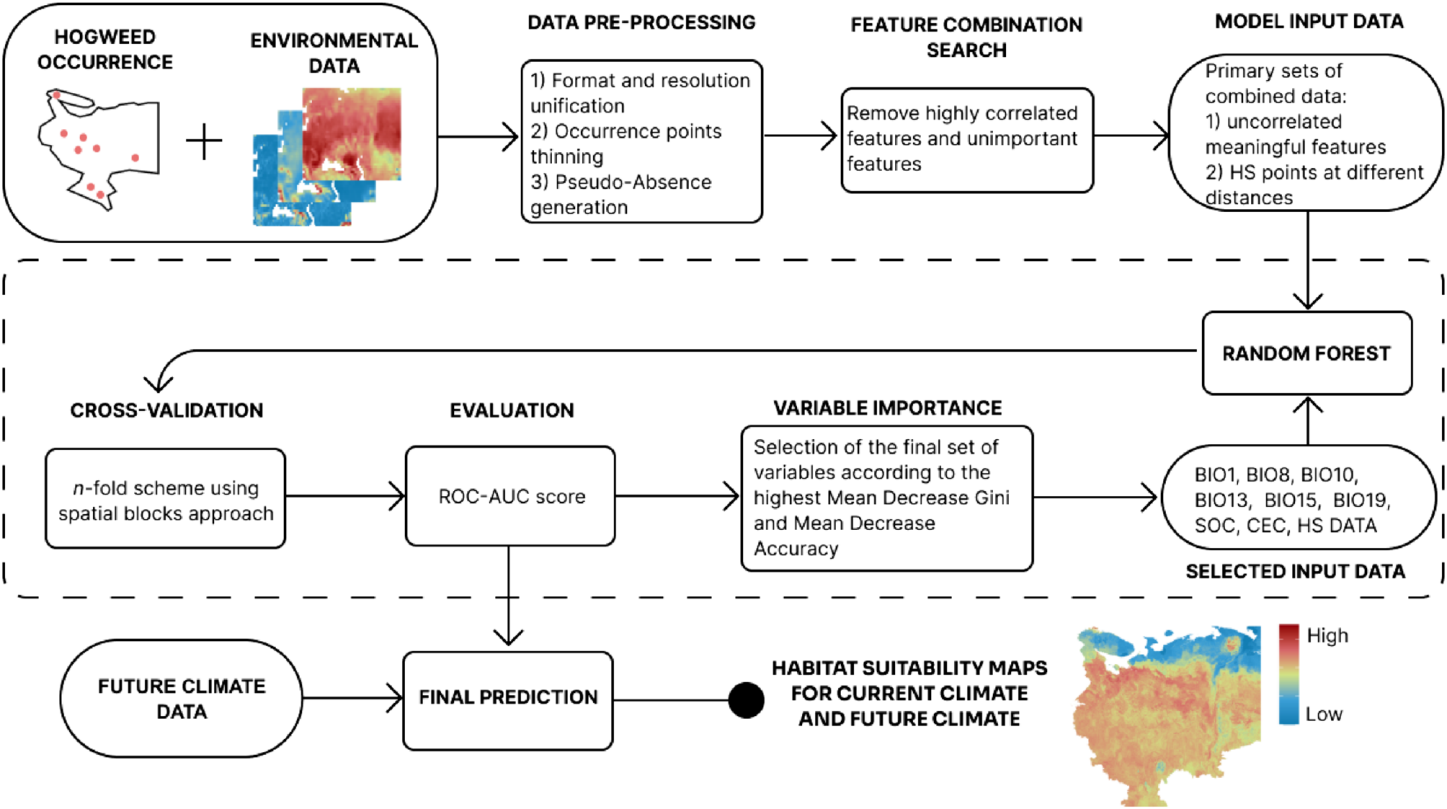
Flowchart of the approach.

While it is extremely difficult to eliminate all growing populations of the invasive species, HS including, the information from the modelling of habitat suitability can aid in prioritizing the management of invaded areas. Precisely, it can help to mark out the territories where the possibility of development of rapidly growing populations poses the largest threat to native species, agriculture, and populated areas. Considering this context use of data from CSP is of particular interest, however, it may have its limitations. In this work large-scale HS distribution modelling is performed. We estimated habitat suitability for the current climate as average from 2000 to 2018 and possible future climate from 2040 to 2060 according to three climate models—BCC-CSM2-MR, CanESM5, and CNRM-CM6-1—in two scenarios, the worst and the best in terms of greenhouse gas emissions (Fig. 1).

The general workflow of the presented research included the following steps: (i) collection of the required data from public sources, (ii) data pre-processing, (iii) feature selection, (iv) model training and validation, (v) receiving of the outputs of the best model, (vi) building the maps, showing the spatial distribution of the occurrence probability (habitat suitability) across the territory of the study, expressed in the range from 0 to 1, for current and possible future climate conditions. Presented methodology and results of HS spread modelling can be used for invasion risk assessment.

## Results

### Optimisation of the occurrence data distribution based on the thinning procedure

**Figure 2:**
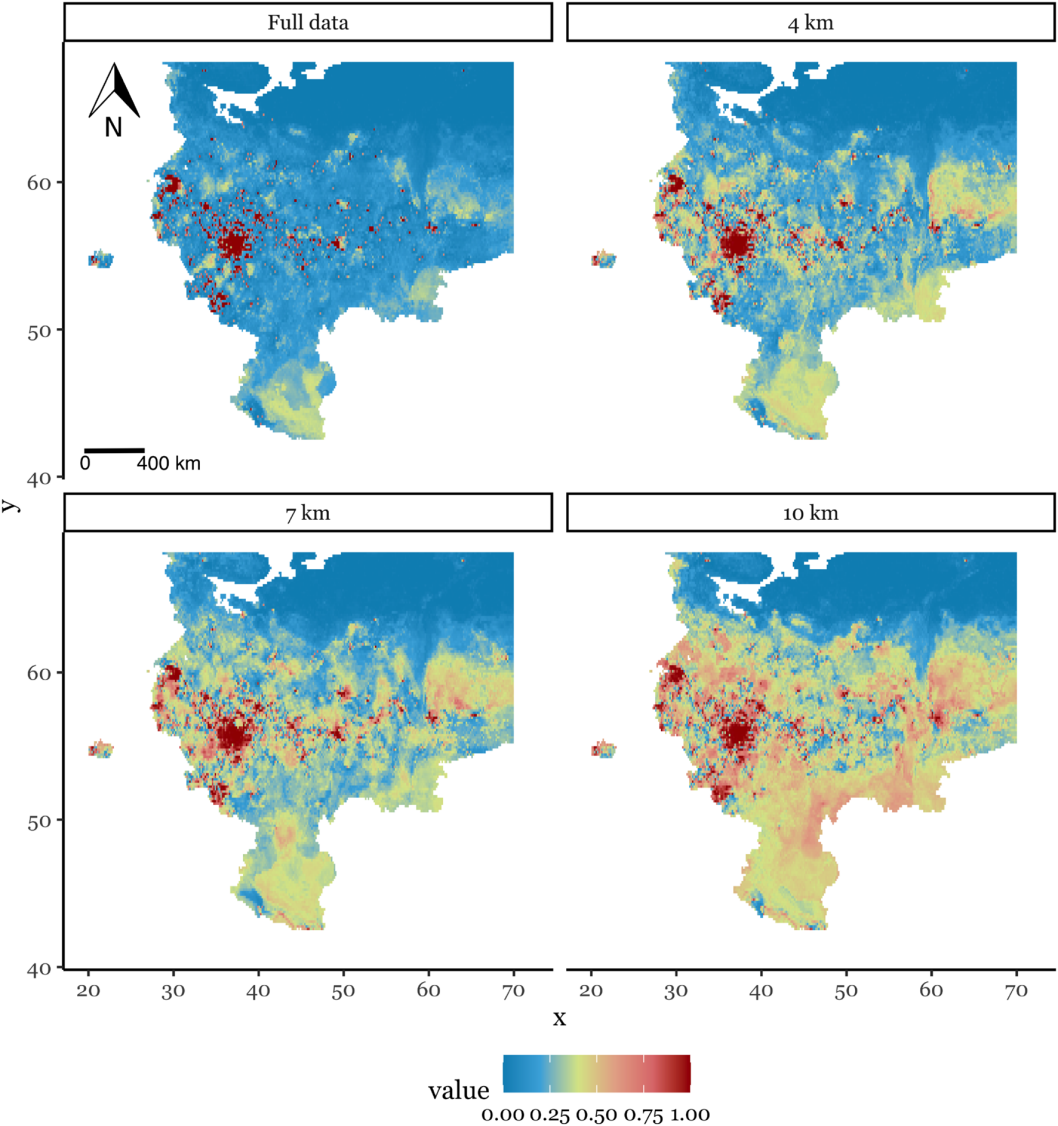
Maps of prediction of possible distribution of HS in current climate conditions using different thinning distances and, consequently, amounts of input points. The quality of prediction varies significantly, while the model built on the full dataset is obviously overfitted.

Ideally, thinning removes the optimal number of records to substantially reduce the effects of sampling bias, as in our case when most of the locations are concentrated in a few places—while simultaneously retaining most of the valuable information. Figure 2 demonstrates the results of model prediction for (1) initial dataset with data collected from all available sources; (2) dataset with thinning distance 4 km, (3) dataset with thinning distance 7 km, (4) dataset with thinning distance 10 km. It is also important to know how the predictors’ distribution would change at the different thinning intervals. In our case, there were no significant differences between distributions’ shapes of environmental features that corresponded to the different thinned data (Fig. S1).

The outputs of models vary significantly depending on the number of points at different input datasets. The ROC-AUC scores of the models built on the complete data, datasets at 10, 7, and 4 km thinning distances are 0.877, 0.83, 0.85, and 0.82 correspondingly (Fig. S4). Modelling results obtained from the complete dataset represent the territory of the study as mostly unsuitable for HS spread, 84% of the territory is characterised by the prediction value of less than 0.25. On the most contrast variant of the model built on the data at 10 km thinning distance, the suitability rose considerably: the percent of territory where the prediction value is above 0.5 increased to 22% compared to 3% in the case of full data, the area of territory under less than 0.25 prediction values is decreased to 31% and 44% at thinning distances of 10 and 7 km respectively. We further need to choose which model to use for the next step of future prediction by finding a reasonable output.

From the results visualised as maps, we can notice, that the model built on the full dataset is overfitted, does not cover northern latitudes, and poorly represent the original habitat located in the Caucasus area. It mostly repeats the points of observation thus the possible distribution of habitat suitability of HS obtained from the full dataset is built in a learn-by-heart manner. On the contrary, the model built on the dataset obtained due to thinning at the distance of 7 km seems to be the most suitable in terms of both prediction results and keeping as much information as possible. Additionally, while we cannot lean on the evaluation scores to support this conclusion, we estimated the variability of prediction values across the territory of the study. It was the highest for the datasets obtained at 10 and 7 km thinning distances. The outputs of the model built on 7 km distance data were more diverse at the 100 km blocks, as was used for spatial cross-validation (Figs. S2, S3).

### Features selected for modelling

To avoid over-fitting because of using redundant variables, an important part of the SDM procedure was choosing the most meaningful set of them, corresponding to the observed HS occurrence. To do this several approaches were combined: search of highly correlated features and estimation of the importance of features by the Mean Decrease Gini (MDG) and the Mean Decrease Accuracy (MDA) scores. Thus, the general workflow consisted of 3 general steps: (1) generation of correlation matrix; (2) estimation of MDG and MDA scores; (3) picking up highly important non-correlated features and choosing the features that have correlates but demonstrate higher importance according to both MDG and MDA scores. The first step of selection includes a search of highly correlated features and the formation of sets of mutually exclusive covariates according to the absolute value of Pearson correlation coefficient greater than 0.8. The correlations are demonstrated in Fig. S5. From the group of bioclimatic variables, the following subset of features demonstrated a high correlations’ coefficients between each other:

- BIO1, BIO6, BIO9, BIO11;
- BIO6, BIO4, BIO7;
- BIO4, BIO7, BIO16;
- BIO5, BIO10;
- BIO16, BIO13;
- BIO13, BIO14, BIO18, BIO17, BIO12.

Then, based on the variable importance results obtained by MDA and MDG, the most important features were selected and included in the core list for the predictions: BIO8, BIO10, BIO13, BIO15, BIO19. Additionally, BIO1 and BIO9 features demonstrated approximately equal importance in corresponding forecastings. Thus, we built different RF models with the core list of features including only BIO9 for the first variant and only BIO1 for the second one. By comparing the results from modelling, BIO1 demonstrated higher importance, so it was included in the final list of features.

Using the same approach, described above, the selection of soil properties was performed. According to the correlation matrix (Fig. S5), soil properties do not have correlation coefficients equal to 0.8 or more in absolute values with bioclimatic variables. However, SOC and Sand content demonstrated a high enough correlation. CF, Silt and Sand did not show high importance in the corresponding analyses. Thus, from the soil features, the final list included only CEC and SOC. Therefore, the following list of features was used to train the algorithm: SOC, CEC, BIO1, BIO8, BIO10, BIO13, BIO15 and BIO19 (Fig. S6).

According to Fig. S6, BIO13 and BIO8 demonstrated the highest importance in predicting HS distribution. Based on MDA, soil properties are considered to be more important compared to MDG. BIO1 and BIO10 demonstrated less importance related to MDA, whereas CEC and BIO19 had the same pattern related to MDG.

### Possible habitat suitability in the future

**Figure 3:**
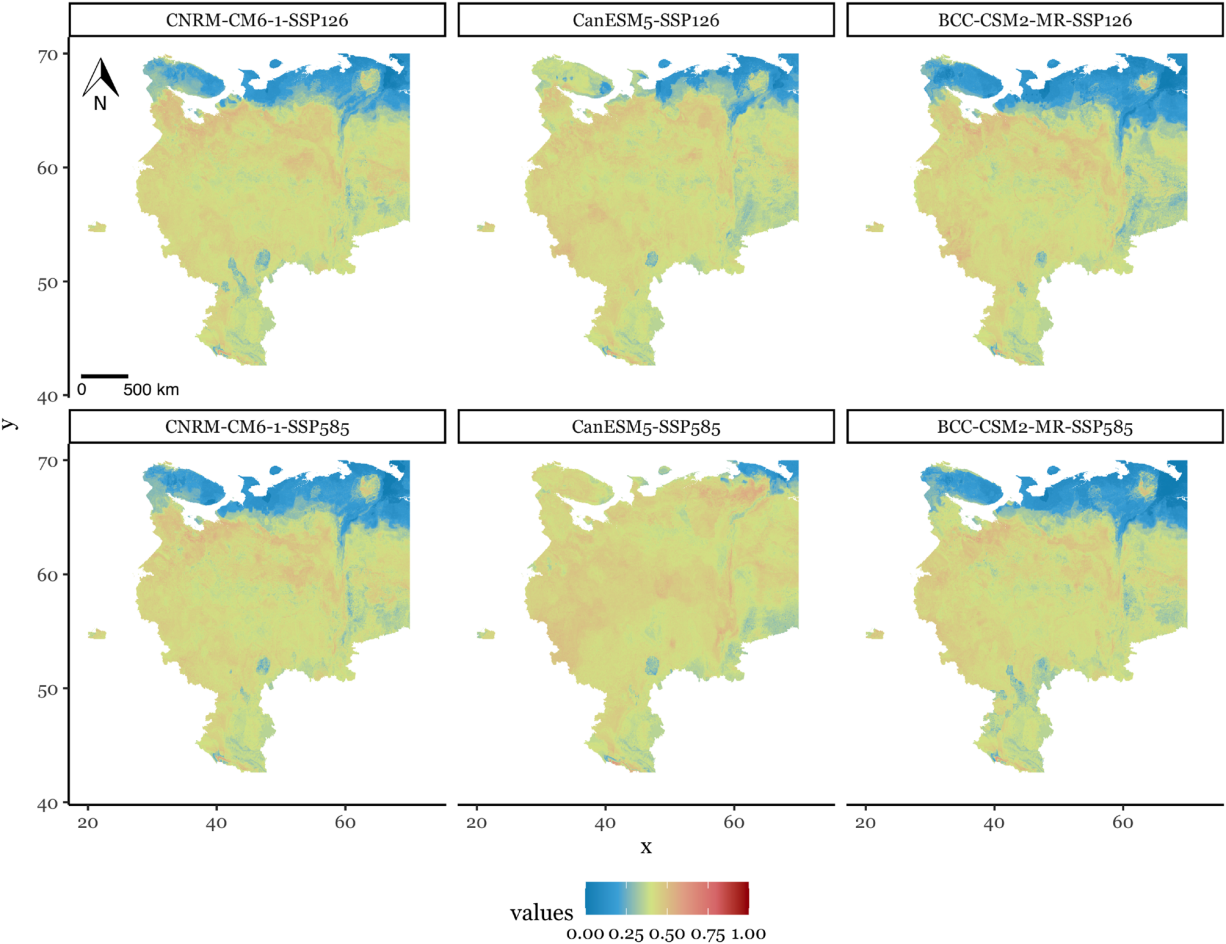
Maps of prediction for possible distribution in future climate conditions on the example of selected global climate models CNRM-CM6-1, CanESM5, BCC-CSM2-MR, in two scenarios of Shared Socioeconomic Pathways—SSP126 and SSP585.

Using the set of environmental predictors obtained in at the feature selection stage, we modelled the possible future spread of HS across the territory of the study. To do this, we estimated the distribution of bioclimatic variables according to the available global climate models. From obtained results, we see that CNRM-CM6-1 and BCC-CSM2-MR show almost identical results in general, as well as between chosen SSP (Fig. 3, Fig. S7). According to them, only 6–8 % of the study territory in the future are characterised by prediction values less than 0.25. The CanESM5 results show a slightly different picture: the percent of the likely unsuitable territory is down even more to 3% at the better SSP126, and to 0.7% at the worst CO$_2$ atmosphere concentration scenario—SSP585.

## Discussion

Sampling bias is a frequent problem in SDM because presence-only data is collected predominantly in more accessible locations^32^. The spatial sampling bias also leads to environmental bias in the data set^33^. Ignoring the problem leads to inaccurate forecasting and can be followed by an inappropriate risk assessment of invasive species and mistakes in conservation planning. The number of methods to tackle the problem of sampling bias is limited. For rare records, a new Poisson point process model is proposed^34^, where environmental effect and sampling bias are explicitly modelled in separate clusters in a framework of quasi-linear modelling. Another example of solving this problem is the incorporation of background (pseudo-absence) points with the same sampling bias as the distribution of occurrence points^33^. If the number of records is enough, spatial filtering is commonly used when some part of occurrence points is discarded. We combined two methods to overcome the sampling bias of HS occurrence points: spatial filtering and the creation of the same effect of sampling bias among pseudo-absence points.

The main limitation of the data thinning method is that we should assume the particular level of sampling bias to proceed to the following modelling, while in reality, it is actually unknown. This is why the search for the most appropriate amount of discarded records for occurrence points is not straightforward. Therefore we used three distances: 4 km, 7 km and 10 km to compare with the initial dataset. The estimates for the current climate (Fig. 2) demonstrate significant differences in predicted habitat suitability that are highly dependent on the thinning process. At the same time, using the initial dataset in forecasting leads to inaccurate results, which demonstrates over-fitting. Extensive results carried out show that spatial filtering improves forecasting, the higher number of discarded points—the higher number of locations that are more suitable for HS. Nevertheless, the big distance of 10 km excludes the largest amount of points which leads to an increase in the uncertainty of the model. Therefore, the distance of 7 km was considered as optimum in our study. However, it must not be forgotten that the suspense of the level of sampling bias is one limitation of our implementation.

The characterization of the possible future expansion of invasive species might be a powerful tool for the highlighting of the importance of conservation management. Research results allow considering possible shifts in the ecosystem components on different levels without actions to be taken.

According to the modelling results the main change between current and possible future distributions is in the noticeable increase of the average possible suitability across the territory from 0.28 in current climate conditions to 0.37–0.4 at SSP126 and to 0.37–0.43 at SSP585 in future climate considering the results of the selected models (Table S1). Moreover, in the future conditions, the territory of study is covered more or less uniformly by the prediction values higher than 0.25—on average 89% is potentially suitable for HS spread, which is two times more, compared to the current climate predictions based on the selected data shape. At the same time, the maximum prediction value in future climate is decreased from 1 to 0.7. The territory covered by prediction values more than 0.25 includes the high latitudes above 60$^{\circ }$, especially in the SSP585 scenario according to the CanESM5 model.

According to established knowledge, the light availability in combination with disturbed upper soil coverage is necessary for the development of HS plants at the early stages^35^. Thus, the conditions of long daylight in northern latitudes during the period of active vegetation might especially favour the plants’ growth in the lighted cover locations with suitable wetness regime. Therefore, HS in future climate might impose a serious danger to the territories  that are currently characterised by low biodiversity and productivity. It can be additionally considered, that the active spread of HS can influence the carbon cycle by reducing biodiversity and changing the structure of soil profile. HS is reported not to form litter^36^, so the structure of microorganisms’ community should shift, while soil properties are expected to degrade due to a decrease of carbon stock^37^.

## Methods

From the popular algorithms, we chose the Random forest model as the most suitable for our case. The data required for predictions can be divided into plant occurrence records and environmental features. Bioclimatic variables and soil properties were selected as the main environmental features. All of the data were obtained from open sources.

### Heracleum Sosnowskiy plant description

*Heracleum sosnowskyi* is a monocarpic perennial plant of the Apiaceae family. The height is up to 3–5 m with a straight stem up to 12 cm in diameter. HS compound steam leaves can reach 150 cm, both long and wide^38^. The blooming period starts in July and continues until the end of September. Plant reproduction is performed by seeds only. The seeds’ depth of germination is reported as mainly in the upper 5 cm down to 15 cm of soil. One plant can produce 10–20,000 seeds^39,40^. Seeds germinate in the early spring, while some have reported that a period of cold stratification for the dormancy break is obligatory for germination development. Suitable conditions for HS include a temperate climate with warm humid summers and cold winters, while it is probably not drought resistant. Plants of HS tend to neutral soils with a pH range from 6 to 7, rich in nutrients, and being reported as nitrophilous, so the eutrophication of the environment favours HS development. HS plants do not tolerate shade conditions in the first growing period.

HS is mostly spread in artificial and semi-natural habitats, including grasslands, pastures, parks, roadsides, agricultural fields, riverbanks or canal sides, and other distributed habitats. Currently, the main pathways of spread include an involuntary entry with soil on vehicles, machinery, footwear or the use of soil as a commodity (as the growing medium rich in organic matter)^39^.

### Study area

**Figure 4:**
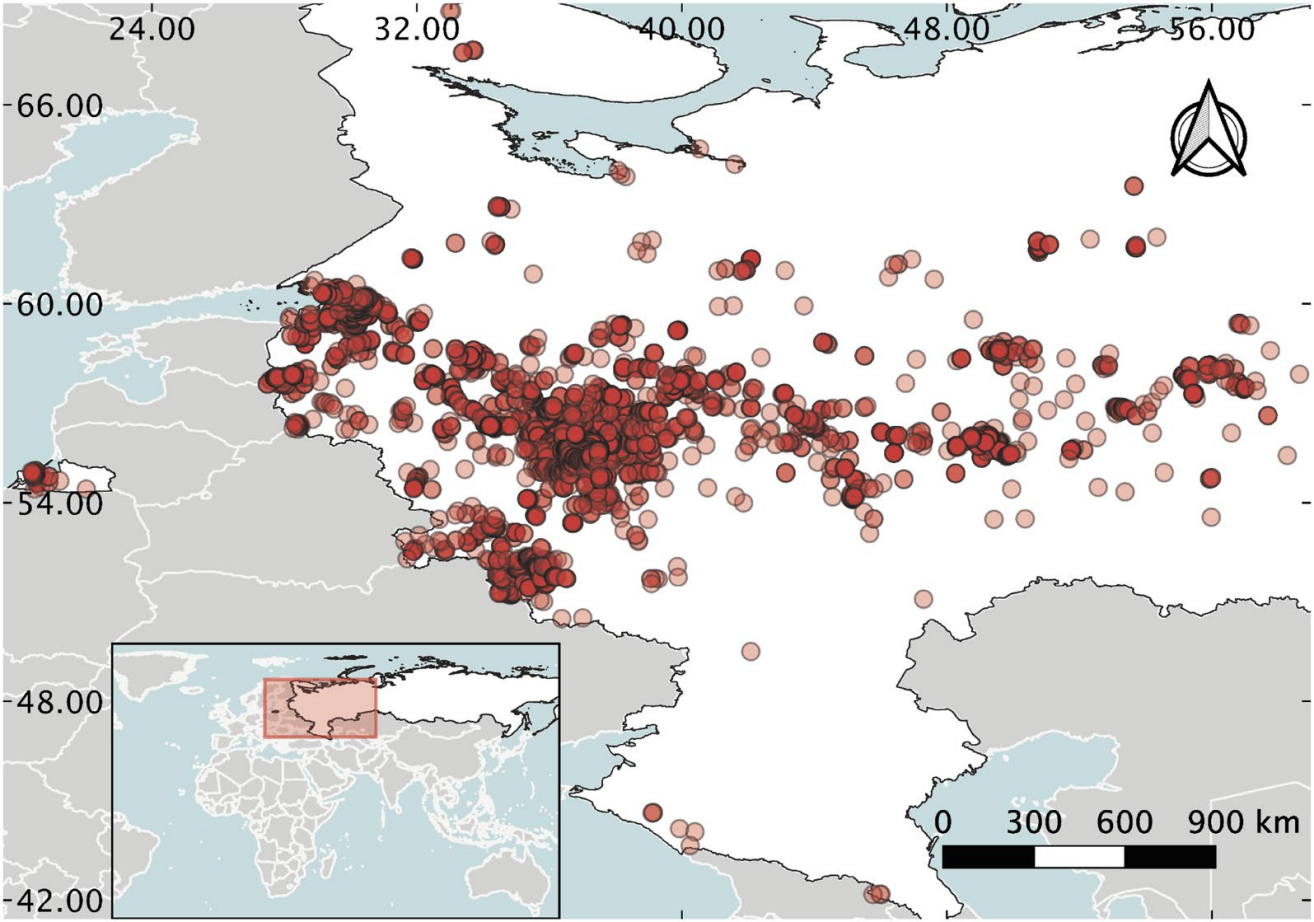
Map of the study area: white colour represents the territory used for prediction, red points correspond to the dataset of HS occurrence, collected from the available sources.

The area for modelling extends from approximately 41$^{\circ }$ to 70$^{\circ }$ N and from 27$^{\circ }$ to 60$^{\circ }$ E, and Kaliningrad region, it equals to approximately 4 mln km^2^ (Fig. 4).

The European part of Russia is the most inhabited part of the country, and it is the home of approximately 80% of the total population of Russia. It includes the East European Plain, Caucasus mountains and Ural mountains, with the predominance of the East European Plain. Environmental characteristics across the territory of study vary significantly. The climate is changing from semi-arid in the south to subarctic in the north, including humid continental climate conditions. Natural vegetation is represented by almost all types of biomes with the prevalence of different types of forests: broadleaf and mixed forests, coniferous forests, and boreal forests (taiga), while the area of arable lands is reported to be approximately 650,000 km^2^^41,42^. The territory is subjected to the constant land-use types and cover changes due to the urbanization and switch of the status of arable lands—i.e. reduction of croplands and development of fallows and forests, and, vice versa, returning of some of them into the cultivation process^43^. The soil cover is represented by the contrast by their physicochemical properties groups, in the northern part of Luvisols, Podzols, Histosols, while of the southern part—by Chernozems, Kastanozems, Solonetz^44^.

### Collection of the input data

#### Plant occurrence data

Plant occurrence coordinates were collected from several publicly available sources related to citizen science projects: the Global Biodiversity Information Facility database^45^, iNaturalist database^46^, and the database of the “Antiborschevik” community^47^. Records were documented by human observation and collected from 2000 to 2021. The overall number of initial occurrence points from combined sources is 7637.

#### Environmental predictors

*Climate data* Modelling was performed for current and future climate conditions at its two scenarios, selected year ranges were 2000–2018 and 2040–2060 respectively.

Climatic variables were collected from the Worldclim database^48^, containing the average seasonal information relevant to the physiological characteristics of species and available at different resolutions. We chose 10 arc-minutes spatial resolution taking into account the size of the studied area. Table 1 provides a short description of the used bioclimatic features, and we refer the reader to the Worldclim project for detailed information on the variables’ calculation.

For the future climate scenarios, we used two Shared Socioeconomic Pathways (*SSPs*)^49^—1-2.6 and 5-8.5, corresponding to the lowest (keeping global mean temperature increase below 2 $^{\circ }$C) and the highest (at the increase of population without technological change) predicted future greenhouse gases emission scenarios. For these data, we took the same resolution (10 arc-minutes) as discussed above.

We used the Equilibrium Climate Sensitivity to select the climate model to model future HS distribution. Equilibrium climate sensitivity (ECS) is defined as the global mean surface air temperature change due to a rapid doubling of carbon dioxide concentrations as soon as the associated ocean-atmosphere-sea ice system reaches equilibrium. As the ECS value increases, the model’s sensitivity to the CO$_2$ concentration in the atmosphere increases. We have chosen CanESM5 model (ECS—5.6), CNRM-CM6-1 model (ECS—4.3) and BCC-CSM2-MR model (ECS—3.0)^50^.

**Table 1:** Description of used bioclimatic variables.

Parameter	Full name
BIO1	Annual mean temperature
BIO2	Mean diurnal range
BIO3	Isothermality
BIO4	Temperature seasonality
BIO5	Max temperature of warmest month
BIO6	Min temperature of coldest month
BIO7	Temperature annual range
BIO8	Mean temperature of wettest quarter
BIO9	Mean temperature of driest quarter
BIO10	Mean temperature of warmest quarter
BIO11	Mean temperature of coldest quarter
BIO12	Annual precipitation
BIO13	Precipitation of wettest month
BIO14	Precipitation of driest month
BIO15	Precipitation seasonality
BIO16	Precipitation of wettest quarter
BIO17	Precipitation of driest quarter
BIO18	Precipitation of warmest quarter
BIO19	Precipitation of coldest quarter

For the future climate scenarios we selected three climate models:

- *BCC-CSM2-MR* Beijing Climate Center climate system model developed in Beijing Climate Center, China Meteorological Administration^51^. Model has horizontal resolution 1.125$^{\circ }$ by 1.125$^{\circ }$.
- *CanESM5* Canadian Earth System Model version 5 developed in Canadian Center for Climate Modelling and Analysis, Canada^52^. Horizontal resolution 2.81$^{\circ }$ by 2.81$^{\circ }$.
- *CNRM-CM6-1* Climate model developed in National Center of Meteorological Research, France^53^. Horizontal resolution 1.4$^{\circ }$ by 1.4$^{\circ }$.

Authors of the WorldClim project prepared historical and future climate data to a uniform spatial (10 arc-minutes) and temporal resolution.

*Soil data* Soil data were downloaded from the SoilGrids database^54^—a system for global digital soil mapping. SoilGrids provides continuous data at several depths of the spatial distribution of soil properties across the globe with selected resolution. It uses a machine learning approach to reconstruct continuous data from 230,000 soil profile observations from the WoSIS (The World Soil Information Service) database and a series of environmental covariates.

From the whole set of the data provided by SoilGrids several properties were chosen for the forecasting: relative percentage of silt (*Silt*, %), sand (*Sand*, %), a volumetric fraction of coarse fragments (*CF*, %), cation exchange capacity (*CEC*, ${\text{cmol}}_{c}/{\text{kg}}$) and soil organic carbon (*SOC*, g/kg) at the depth 5–15 cm, where the HS seeds are assumed to be located. These variables are expected to be more stable over time than bioclimatic predictors; thus, chosen soil properties could be implemented for the future time the same as in the present.

### Data pre-processing

All the data were transformed to the ASCII format by R script and using software DIVA-GIS following the tutorial for the preparation of WorldClim files for use in SDM (http://www.lep-net.org/wp-content/uploads/2016/08/WorldClim_to_MaxEnt_Tutorial.pdf) with unified selected resolution 340 sq.km.

#### Optimization of the occurrence points amount

The general problem in using the available data collected from the databases of the citizen science projects is that the points of observation are distributed non-uniformly. For instance, the frequency of the records depends on the density of the population directly. The spatial filtering of the data (reducing the number of points) can be performed to reduce the sampling bias^55^. We prepared three datasets with a distance between points of 4, 7 and 10 km with 2402, 1846 and 1504 occurrence points correspondingly filtering the initial dataset. For the thinning step *thin()* function was used within the R package *spThin* with 100 iterations for each of chosen thinning distances. To understand how much data we could lose, we used the analysis of feature distribution and evaluated the general fairness of the model performance.

#### Pseudo-absence generation

Due to the availability only of the presence points, it is important to generate the absence points for further implementation of the selected algorithm. Although the generation of pseudo-absence points in SDM research is a widespread solution, a closer look at the literature reveals several gaps and shortcomings. Since the raw dataset of the HS distribution demonstrates strong sampling bias, the generation of pseudo-absence points using the usual ‘random’ strategy can aggravate the sampling bias problem. Thus, the combination of the ‘disk’ and ‘random’ strategies was applied for the generation of the pseudo-absence points using the *biomod* R package^17^.

- The ‘disk’ strategy is established on the geographic distance works as separation from truth presence and possible absence points. The optimal geographic distance for *HS* was chosen as 25 km. This distance was chosen empirically by trial-and-error. We started with 18 km (because the size of the cell is   9–18 km depending on location) and finished with 50 km. Using distances such as 30–50 km lead to a positive spatial autocorrelation. Thus, we decided to set 25 km which finally provided both optimal model performance and reduced spatial autocorrelation.
- The second part of the generation was based on the ‘random’ strategy with filtration: according to the different range of climate conditions on the territory of Russia, there are several places where *HS* is not detected, thus not growing. The selection of unsuitable places for *HS* related to the north of Russia, where it is might be too cold for plant species. From all amount of randomly generated generated points we selected points with condition latitude $> 64^{\circ }$, according to tundra board line.

#### Features selection procedure

To avoid over-fitting and to choose the most conscientious set of parameters for final modelling, two approaches were combined. We searched features that are not correlated with others by a selected threshold is equal to 0.8 in absolute values^56^ and estimated variable importance using the Mean Decrease Gini (*MDG*) and the Mean Decrease Accuracy (*MDA*) as the result of modelling on enumerated parameters’ combinations. MDG score is related to the homogeneity of the nodes and leaves coefficient. With the rise of the MDG score the importance of the corresponding feature is also increasing. MDA describes how much accuracy decrease by removing the feature. We selected the most important features according to the MDG and MDA scores by the highest values of both metrics using a sequential search from an initial set of variables.

### Modelling approach

#### Random forest

Choosing the appropriate method for creating the tool for accurate SDM is crucial because the overall performance could vary dramatically, depending on the selected model and particular use case. There is a limited amount of acceptable machine learning methods that can be used in SDM. Several popular methods demonstrated high performance in modelling on large areas: GBM, RF, and GLM. In particular, for modelling and prediction of the potential distribution of invasive species, GLM and RF were used^57^. We decided to use RF because this model was successfully implemented for solving a variety of tasks such as predictions of animal and plant distributions, and also was used for making predictions on a large territory^58^. The other important advantage that should be noticed is the straightforward interpretability of RF, which means that it is possible to evaluate the impact of each environmental parameter on the occurrence of the invasive species.

#### Approach to the cross-validation of the model

A unique approach for the model calibration is needed to reduce spatial autocorrelation caused by the absence of a strict sampling design. In our case, the data was split into training and testing folds using the spatial blocks technique in a scheme of 13-fold cross-validation. Random spatial splitting was performed 20 times to calibrate the model, with a distance between blocks set as 100 km. To calibrate the model we used a spatial blocks approach with random type from R package *blockCV*.

#### Evaluation of the model performance

To evaluate the performance of the model a classic approach for ecology was used—Area Under Curve (*AUC*) or Receiver operating characteristic (*ROC*), related to the independent threshold techniques^16^. The principle of methods lies in the standard confusion matrix, where rows and columns represent actual and predicted classes. The construction of ROC curves uses all possible thresholds to obtain different confusion matrices which leads to the reproduction of the curve with two-dimensional space: (1) on y-axis is True Positive Rate (sensitivity, recall); (2) on x-axis is False Positive Rate (equal to 1 − specificity). In our case true positive (*TP*, sensitivity) rate means that predicted places where HS grows correspond to actual. Similarly, true negative rate (*TN*, specificity) indicates correctly classified locations as absence points. In contrast, the missteps when the model predicted places as presence points for plants that are incorrect are False Positive, *FP*, and places where HS is absent, according to the model, while this is not true are recognised as False Negative, *FN*.

## Supplementary Information

Supplementary Information.

## Data Availability

For the data, preprocessing and modelling details to reproduce the calculations, we refer the reader to the repository of the project https://github.com/heracleumsosnowskyi/Heracleum_Sosnowskyi.
