# Supplementary material for: Large-scale forecasting of Heracleum sosnowskyi habitat suitability under the climate change on publicly available data
Source: Sci Rep. 2022 Apr 12;12:6128. doi: 10.1038/s41598-022-09953-9 (PMC9005721; doi:10.1038/s41598-022-09953-9)
Supplement: Supplementary file 1 — Supplementary Information. [file 41598_2022_9953_MOESM1_ESM.pdf]

8 Supporting Information

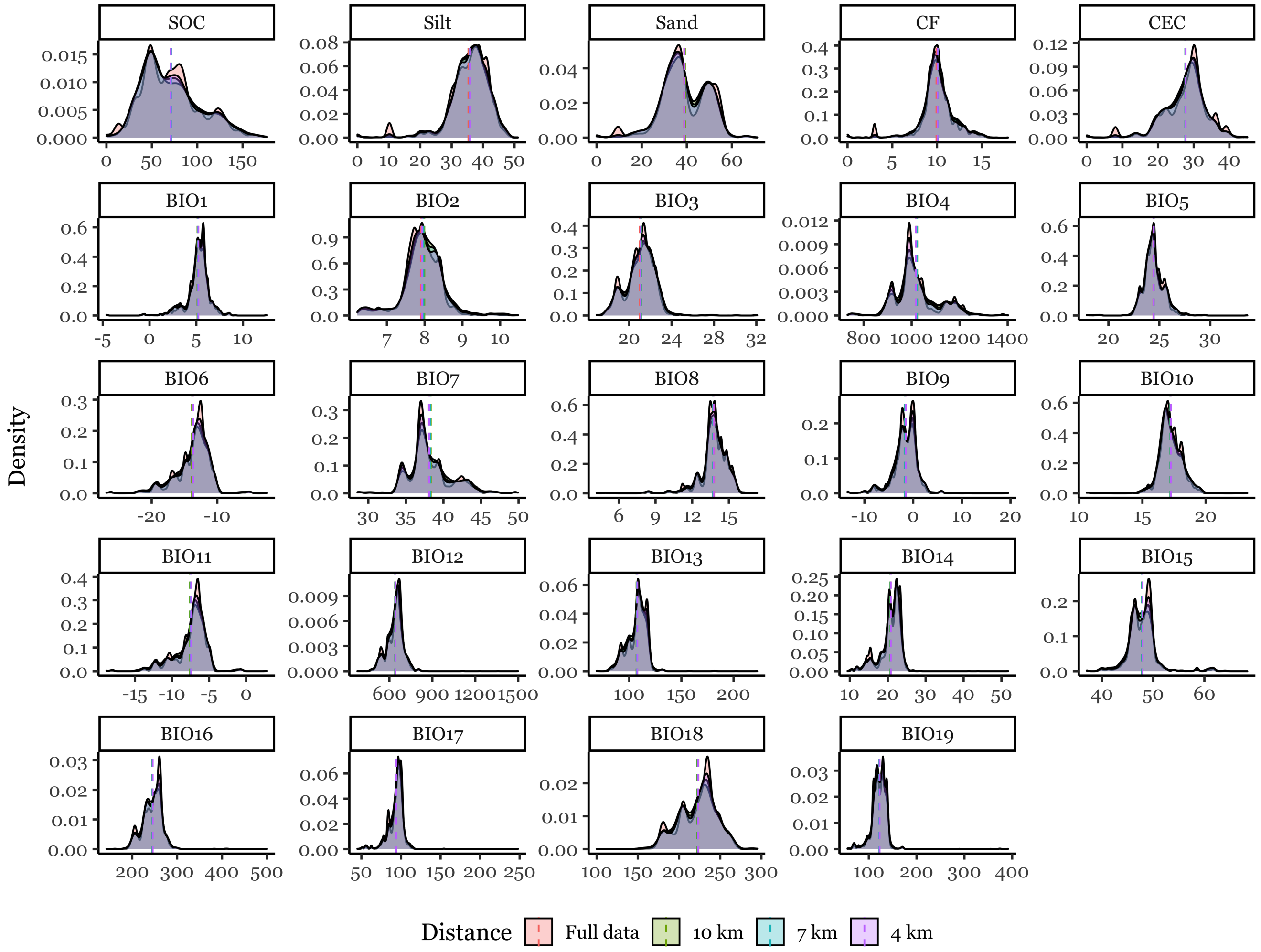

Figure S1. Density plots of environmental features for full dataset and datasets with reduced amounts of points at the chosen thinning distances: 10, 7 and 4 km. Dashed lines represent mean values of the feature for each of datasets. The density plots of all datasets are overlapped so quite similar with no difference between means.

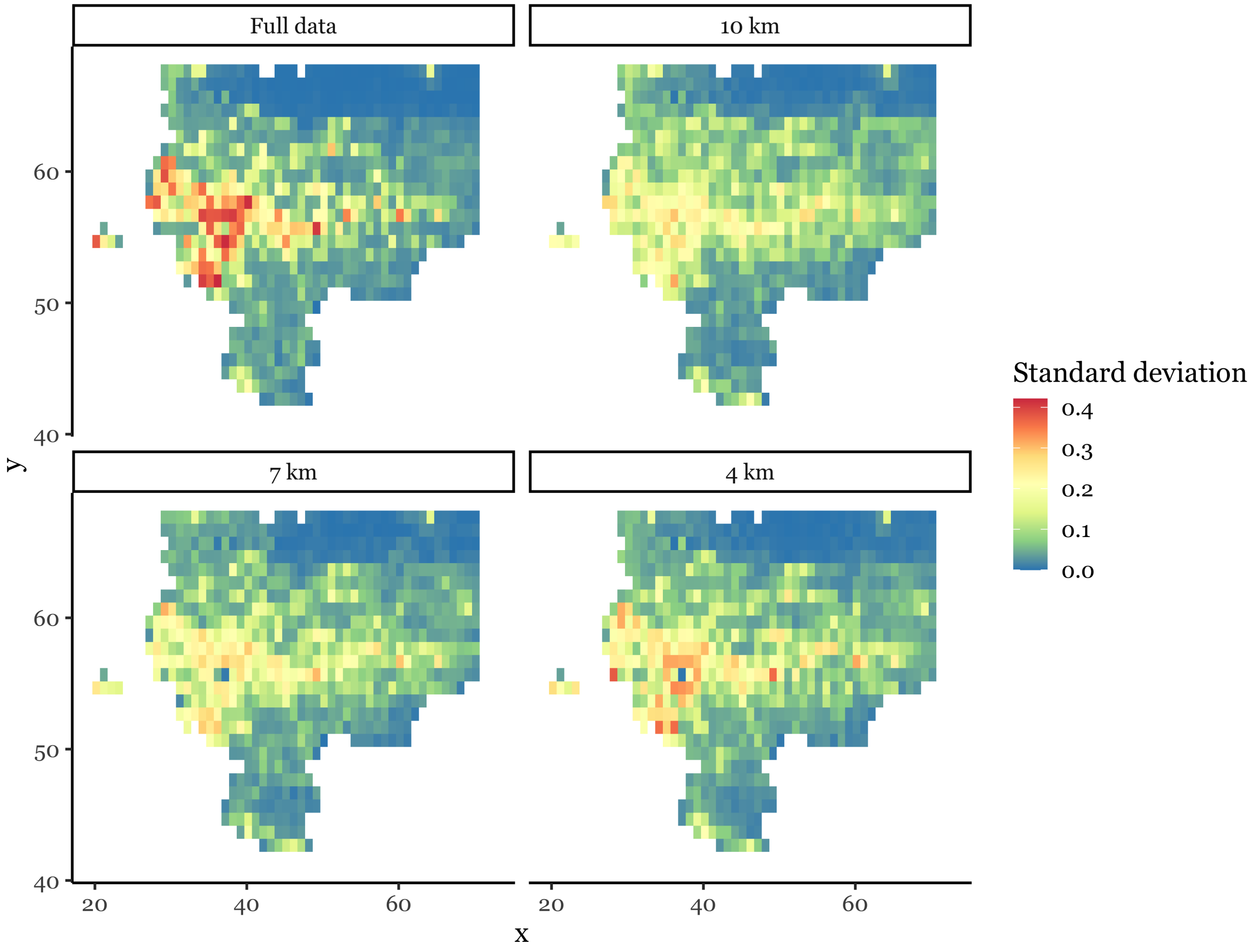

Figure S2. Prediction maps aggregated by standard deviation obtained on the full dataset and datasets with reduced amount of points at chosen distances for the current climate conditions. Aggregation was performed from initial resolution to blocks 100x100 km^2.

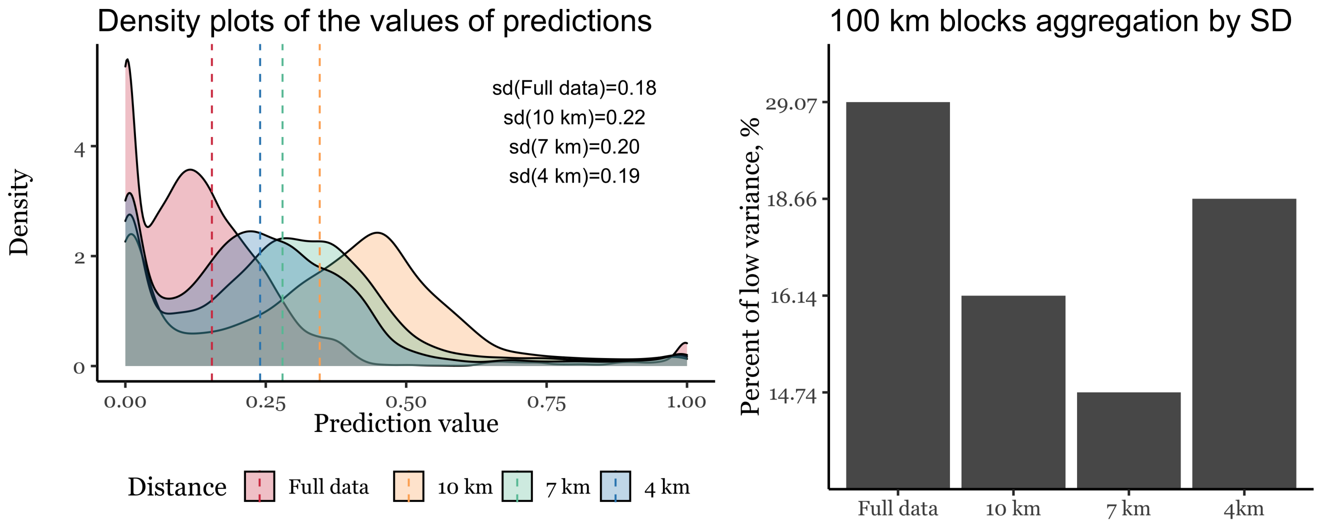

Figure S3. Characteristics of the model outputs obtained on the full dataset and datasets with reduced number of points at chosen distances for the current climate: density plots with means represented by dashed lines (left) and percent of the data with low variance (right) estimated on the normalized standard deviation (SD).

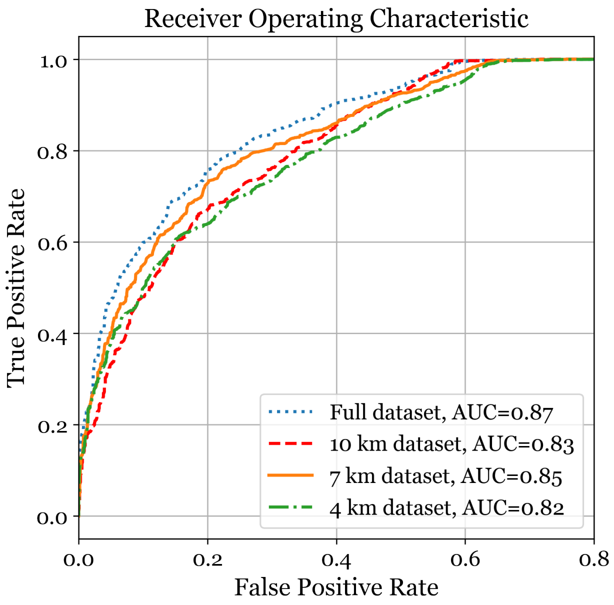

Figure S4. The figure indicates the ROC-AUC curve of the models built on the complete dataset, datasets at 10, 7 and 4 km thinning distances. The dataset at 7 km thinning attain a better ROC-AUC score than 10 and 4 km.

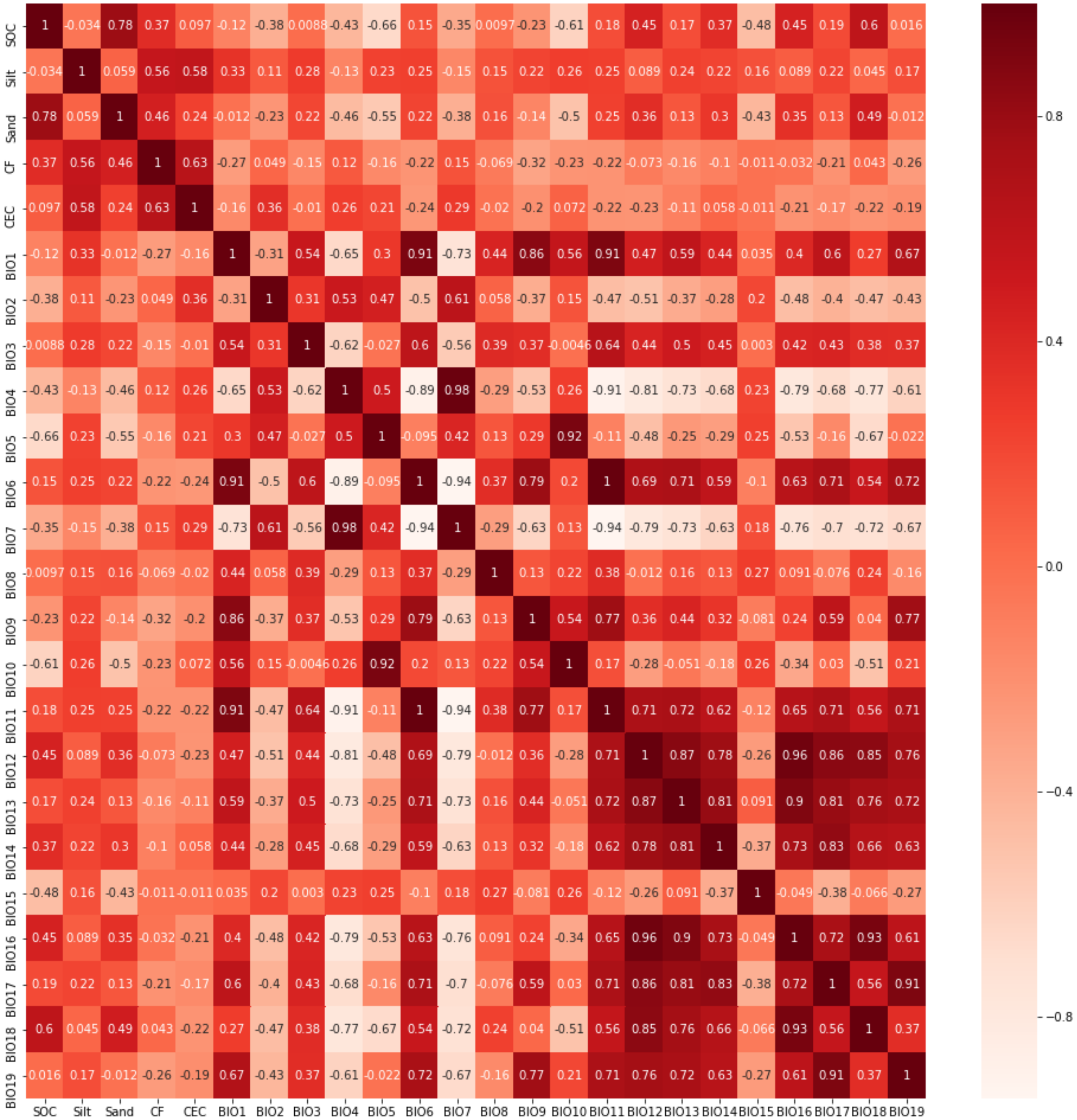

Figure S5. Correlation matrix of all features

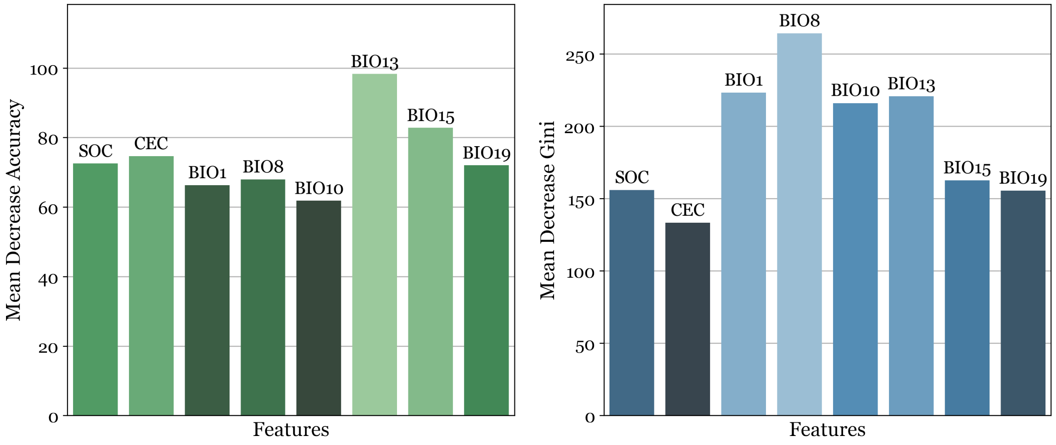

Figure S6. The figure shows feature importance calculated by mean decrease accuracy and mean decrease Gini of variables selected for modelling.

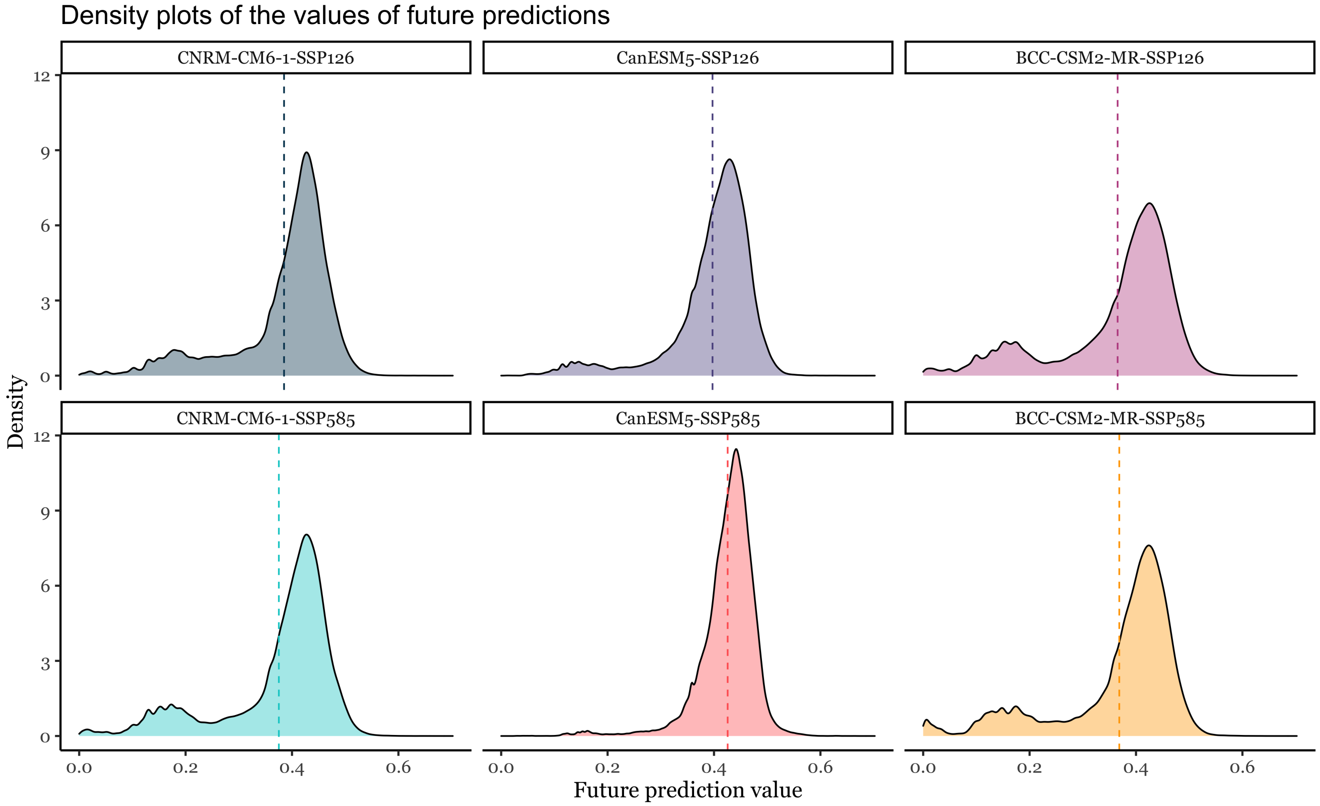

Figure S7. Density plots of future predictions distributions with means represented by dashed lines. It can be seen that distributions look quite similar apart from the appearance of left-located peaks in cases of CNRM-CM6-1 and BCC-CSM2-MR models

Table S1. Characteristics (mean values – Mean, standard deviation – SD, minimum – MIN, maximum – MAX, and percent of results more than 0.4 – PV>0.4) of the distributions of future predictions of used climate models:

	SSP-126			SSP-585		
Parameter	CNRM	Can	BCC	CNRM	Can	BCC
Mean	0.39	0.4	0.37	0.38	0.43	0.37
SD	0.09	0.08	0.11	0.1	0.05	0.11
MIN	0	0.01	0	0	0.03	0
MAX	0.66	0.69	0.7	0.7	0.67	0.65
PV> 0.4	59.74	61.51	51.45	56.33	78.7	53.31
